# Hydrogen Sulfide Ameliorates Blood-Spinal Cord Barrier Disruption and Improves Functional Recovery by Inhibiting Endoplasmic Reticulum Stress-Dependent Autophagy

**DOI:** 10.3389/fphar.2018.00858

**Published:** 2018-08-28

**Authors:** Haoli Wang, Yanqing Wu, Wen Han, Jiawei Li, Kebin Xu, Zhengmao Li, Qingqing Wang, Ke Xu, Yanlong Liu, Ling Xie, Jiang Wu, Huacheng He, Huazi Xu, Jian Xiao

**Affiliations:** ^1^Department of Orthopaedics, The Second Affiliated Hospital and Yuying Children’s Hospital of Wenzhou Medical University, Wenzhou, China; ^2^Molecular Pharmacology Research Center, School of Pharmaceutical Science, Wenzhou Medical University, Wenzhou, China; ^3^The Institute of Life Sciences, Wenzhou University, Wenzhou, China

**Keywords:** hydrogen sulfide (H_2_S), spinal cord injury (SCI), blood-spinal cord barrier (BSCB), ER stress, autophagy

## Abstract

Spinal cord injury (SCI) induces the disruption of blood-spinal cord barrier (BSCB), which elicits neurological deficits by triggering secondary injuries. Hydrogen sulfide (H_2_S) is a gaseous mediator that has been reported to have neuroprotective effect in the central nervous system. However, the relationship between H_2_S and BSCB disruption during SCI remains unknown. Therefore, it is interesting to evaluate whether the administration of NaHS, a H_2_S donor, can protect BSCB integrity against SCI and investigate the potential mechanisms underlying it. In present study, we found that SCI markedly activated endoplasmic reticulum (ER) stress and autophagy in a rat model of complete crushing injury to the spinal cord at T9 level. NaHS treatment prevented the loss of tight junction (TJ) and adherens junction (AJ) proteins both *in vivo* and *in vitro*. However, the protective effect of NaHS on BSCB restoration was significantly reduced by an ER stress activator (tunicamycin, TM) and an autophagy activator (rapamycin, Rapa). Moreover, SCI-induced autophagy was remarkably blocked by the ER stress inhibitor (4-phenylbutyric acid, 4-PBA). But the autophagy inhibitor (3-Methyladenine, 3-MA) only inhibited autophagy without obvious effects on ER stress. Finally, we had revealed that NaHS significantly alleviated BSCB permeability and improved functional recovery after SCI, and these effects were markedly reversed by TM and Rapa. In conclusion, our present study has demonstrated that NaHS treatment is beneficial for SCI recovery, indicating that H_2_S treatment is a potential therapeutic strategy for promoting SCI recovery.

## Introduction

The blood-spinal cord barrier (BSCB) is primarily composed of a specialized system of endothelial cells (ECs) and accessory structures, including the basement membrane, astrocytic end feet processes and pericytes. BSCB forms a tight structure owing to the well-developed tight junctions (TJs) proteins and adherens junctions (AJs) proteins that block the entry of molecules into the spinal cord (Bartanusz et al., 2011; Zheng et al., 2017). The degradation of TJs and AJs proteins causes BSCB destruction and consequently increases BSCB permeability after spinal cord injury (SCI) (Lee J.Y. et al., 2012). BSCB disruption allows blood cells to infiltrate the injured parenchyma and aggravates secondary injuries, such as local edema, ischemia, focal hemorrhage, and inflammation (Fang et al., 2015; Li et al., 2017; Lee et al., 2018). Thus, targeting the BSCB disruption is considered as a primary therapeutic approach for SCI.

Hydrogen sulfide (H_2_S) is a gaseous molecule with a rotten egg smell that has been recognized as having a toxic effect. Recent studies have also demonstrated that H_2_S plays a crucial role in physiological and biological effects (Abe and Kimura, 1996; Fiorucci et al., 2006; Martelli et al., 2012). As an almost ubiquitous novel gasotransmitter, H_2_S plays an important role in oxidative stress (Xie et al., 2017b), inflammation (Magierowski et al., 2017) and cardiovascular protection (Calvert et al., 2009). Moreover, it has been reported that H_2_S has neuroprotective effect against oxygen-glucose deprivation (OGD)-induced neuron injury (Yu et al., 2017). Recently, H_2_S has been demonstrated to ameliorate homocysteine-induced brain blood barrier disruption in mice (Kamat et al., 2016). However, it is unclear whether H_2_S plays a role in protecting BSCB integrity after SCI.

Under physiological conditions, the aggregation of misfolded proteins in the endoplasmic reticulum (ER) disrupt ER function, and this process is known as ER stress (Lai et al., 2007). Recent studies have reported that ER stress plays a vital role in a range of neurological diseases, including cerebral ischemia, neurodegeneration disorders and SCI (Gong et al., 2017; Mercado et al., 2018; Wu et al., 2018). Previous studies showed that various cells, such as neurons, oligodendrocytes and astrocytes, could trigger ER stress after SCI (Kuang et al., 2010; Mecha et al., 2012; Wu et al., 2016). Inhibiting ER stress markedly protects neurons against SCI (Acioglu et al., 2016), but the role of ER stress in BSCB disruption after SCI is not completely defined. Increasing evidences have shown that H_2_S is involved in regulating ER homeostasis, and one study indicated that H_2_S can reduce ER stress and apoptosis in cigarette smoke-treated bronchial epithelial cells (Lin et al., 2017). Here, we try to explore whether reduction of ER stress is involved in the protective effect of H_2_S on BSCB restoration after SCI.

Autophagy, a lysosome-dependent self-degradation process, is a vital pathway for the maintenance of cellular homeostasis under both pathological and physiological conditions in CNS (Yang and Klionsky, 2010; Mizushima and Komatsu, 2011). A previous study suggests that autophagy are activated to remove dysfunctional proteins caused by ER stress, indicating that ER stress may be a trigger for the activation of autophagy (Ding and Yin, 2008; Rzymski et al., 2010). In addition, a large number of studies have shown that autophagy plays an important role in acute injuries, such as SCI and traumatic brain injury (He et al., 2016; Bai et al., 2017; Xie et al., 2017a). Recently, some studies have indicated that H_2_S is associated with autophagy. It has showed that H_2_S inhibits autophagy by upregulating the PI3K/AKT pathway during therapy for myocardial fibrosis (Xiao et al., 2016). Additionally, H_2_S is involved in restoring cardioprotection from post-conditioning by increasing autophagy (Xiao et al., 2015; Chen et al., 2016). However, evidences concerning the role of autophagy during H_2_S-mediated protection against BSCB disruption after SCI have not been reported, and the relationship between autophagy and ER stress during H_2_S treatment for SCI remains unclear.

In the present study, we had investigated the effect of H_2_S on SCI recovery *in vivo* and *in vitro*, and discovered that H_2_S alleviated the BSCB disruption and permeability, consequently improved functional recovery after SCI. Mechanistic studies had also demonstrated that H_2_S prevent the loss of TJ and AJ proteins, including P120, β-catenin and Occludin, *in vivo* and in OGD-treated ECs. Additionally, we had further explored the role of ER stress and autophagy during H_2_S treatment for SCI.

## Materials and Methods

### Animals and Drug Administration

Eight-week-old adult female Sprague-Dawley rats (200–220 g, *n* = 80) were acquired from the Animal Center of the Chinese Academy of Sciences (Shanghai, China). The animals were housed under a 12 h light/dark cycle at 21–23°C and given *ad libitum* access to food and water. The protocol for the care and use of animals conformed to the guidelines from the National Institutes of Health. All experiments were approved by the Laboratory Animal Ethics Committee of Wenzhou Medical University. For drug administration, NaHS (Sigma-Aldrich, St. Louis, MO, United States) was dissolved in phosphate-buffered saline (PBS). The rats were intraperitoneally (i.p.) injected with NaHS (5.6 mg/kg) 30 min before SCI and were injected with the same dose of NaHS daily for 7d. The ER stress activator tunicamycin (TM) (10 μg/kg), the ER stress inhibitor 4-phenylbutyric acid (4-PBA) (100 mg/kg), the autophagy activator rapamycin (Rapa) (0.5 mg/kg) and the autophagy inhibitor 3-methyladenine (3-MA) (2.5 mg/kg) were i.p. injected immediately after SCI. TM, 4-PBA, Rapa, and 3-MA were purchased from Sigma (Sigma-Aldrich, St. Louis, MO, United States). The control group received the equivalent volume of saline for the same duration. Every effort was made to minimize the pain and discomfort of the animals.

### Induction of Spinal Cord Injury Rat Model

To induce a SCI model, the rats were anesthetized with 10% (w/v) choral hydrate (3.6 mL/kg, i.p.). The skin and muscles adjacent to the spinous processes were incised to expose the vertebral column, and a laminectomy was then performed at the T9 level. The exposed spinal cord was subjected to a moderate crushing injury using a vascular clip (30 g force, Oscar, China) for 1 min (Zheng et al., 2016). The sham group underwent identical procedures but sustained no impact injury. Postoperative monitoring included manual emptying of the bladder twice a day. All rats showed no remarkable side effects resulting from the drug treatment.

### Evans Blue Dye Assays

The integrity of BSCB was evaluated using Evans blue (EB) dye. The rats were injected with 2% EB dye (2 mL/kg) 1d after SCI by intravenous tail injection. Two hour after the injection, the rats were anesthetized and sacrificed via intra-cardiac perfusion with 0.9% saline. Then Spinal cord tissues were stripped and captured.

### Locomotion Recovery Assessment

The Basso Beaattie Bresnahan (BBB) scale and footprint analysis were used to analyze locomotion recovery (Li et al., 2018). The scores ranged from 0 (complete paralysis) to 21 (normal locomotion). The locomotor activity of the rats was evaluated in an open experimental field for 5 min. The animals were evaluated 0, 1, 3, 5, 7, and 14d after surgery. The footprint analysis was performed by dipping the rat’s posterior limb in a red dye and the fore limb in a blue dye. Outcome measures were obtained by five independent investigators who were blinded to the experimental conditions.

### Cell Culture and Oxygen-Glucose Deprivation

HUVECs were expanded and maintained in endothelial cell medium (ECM, ScienCell, Carlsbad, CA, United States) supplemented with 1% ECGS (ScienCell, Carlsbad, CA, United States), 5% FBS (ScienCell, Carlsbad, CA, United States), and antibiotics (100 μg/mL streptomycin and 100 U/mL penicillin, ScienCell, Carlsbad, CA, United States) in a humidified atmosphere of 5% CO_2_ and 95% air at 37°C. The forth generation of cells was used in our study. For the OGD treatment, the cells were refreshed with glucose-free DMEM containing TM (3 μM) or Rapa (100 nM) and immediately placed in a sealed chamber loaded with a mixed gas of 5% CO_2_ and 95% N_2_ for 6 h. The cells were pre-treated with NaHS (100 μM) for 2 h before OGD stimulation. All experiments were performed in triplicate.

### Western Blotting Analysis

Spinal cord tissue samples were extracted 1d after surgery and immediately stored at -80°C for western blotting. Briefly, the tissues were lysed using RIPA buffer (0.5% sodium deoxycholate, 1% Triton X-100, 1 mM EDTA, 1 mM PMSF, 10μg/mL leupeptin, 20 mM Tris-HCl, pH 7.5, and 150 mM NaCl). *In vitro*, the cells were washed twice with PBS and lysed in lysis buffer (0.1% SDS, 1% sodium deoxycholate, 1% Nonidet P-40, 25 mM Tris-HCl, pH 7.6, and 150 mM NaCl). Tissue and cell lysates were centrifuged at 12,000 rpm for 10 min at 4°C, and the supernatant was obtained for a protein assay. Protein concentrations were quantified with BCA reagents (Thermo). An 80μg (*in vivo*) or 40 μg (*in vitro*) aliquot of protein was separated by SDS-PAGE and transferred onto a PVDF membrane (Bio-Rad). The membrane was blocked with 5% (w/v) non-fat milk (Bio-Rad) in TBST (Tris-buffered saline with 0.1% Tween-20) for 2 h at room temperature, and the membranes were then incubated with the following primary antibodies: GAPDH (1:10000 Bio-world), β-catenin (1:1000, Abcam), P120 (1:1000, Abcam), Occludin (1:1000, Abcam), phosphor-JNK (1:200 Santa Cruz), JNK(1:200 Santa Cruz), GRP78 (1:1000, Abcam), PDI (1:1000, Abcam), ATF6 (1:1000 Abcam), CHOP (1:1000 CST), Cle-caspase 12 (1:1000 Abcam), phosphor-mTOR(1:1000 Abcam), mTOR (1:1000 Abcam), ATG7(1:1000 Abcam), ATG5(1:1000 Novus), Beclin1 (1:1000, Abcam), and LC3B I/II (1:1000, Novus) at 4°C overnight. The membranes were washed with TBST three times and incubated with the secondary antibodies for 1 h at room temperature. Signals were visualized by a Chemi DocXRS+Imaging System (Bio-Rad). We analyzed the bands by using the Quantity-One software.

### Immunofluorescence Staining

The rat tissues were fixed in 4% paraformaldehyde (PFA) in PBS for 24 h, embedded in paraffin, and then sectioned into 5 μm slices. The sections were deparaffinized, rehydrated and washed three times for 15 min in PBS. For immunofluorescence staining *in vitro*, the cells were inoculated on cover glasses and fixed for 15 min using 4% PFA. Next, the cells were washed in PBS three times for 2 min each. The sections and cells were incubated with 5% BSA in PBS containing 0.1% Triton X-100 at 37°C for 30 min. Then, the sections were incubated overnight at 4°C with the following antibodies: P120 (1:250 Abcam), β-catenin (1:300, Abcam), Occludin (1:200, Abcam), PDI (1:200, Abcam), and LC3B (1:500, Novus). The sections were washed four times with PBS and incubated with AlexaFluor 488 donkey anti-rabbit/mouse secondary antibodies (1:1000) for 1 h at 37°C. The sections were rinsed four times, incubated with DAPI for 10 min, washed with PBS and sealed with a coverslip. All images were captured on a confocal fluorescence microscope (Nikon, A1 PLUS, Tokyo, Japan).

### TUNEL Assay

DNA fragmentation was detected using an *In Situ* Cell Death Detection Kit (Roche, South Francisco, CA, United States), and TUNEL staining was performed 1d after SCI. The sections were deparaffinized and rehydrated. Then, these sections were treated in a 20 μg/ml proteinase K working solution for 20 min at 37°C. The sections were washed three times in PBS and incubated with TUNEL reaction mixture in a dark humidified chamber for 1 h at 37°C. Afterward, the sections were rinsed with PBS and treated with DAPI for 10 min at room temperature. Positive control was obtained with 10U/mL DNase I buffer for 10 min at room temperature before incubation with the TUNEL reaction mixture. Negative control was incubated with the TUNEL reagent without the TdT enzyme. Positive cells were observed under a confocal fluorescence microscope (Nikon, A1 PLUS, Tokyo, Japan) and analyzed by using Image J software.

### Statistical Analysis

All data were expressed as the mean ± standard error of the mean (SEM) from at least three independent experiments. Statically significant differences were evaluated using one-way analysis of variance (ANOVA) with Dunnett’s *post hoc* test. Differences were considered to be statistical significance when *P* value < 0.05.

## Results

### SCI Activates ER Stress and Autophagy

**FIGURE 1:**
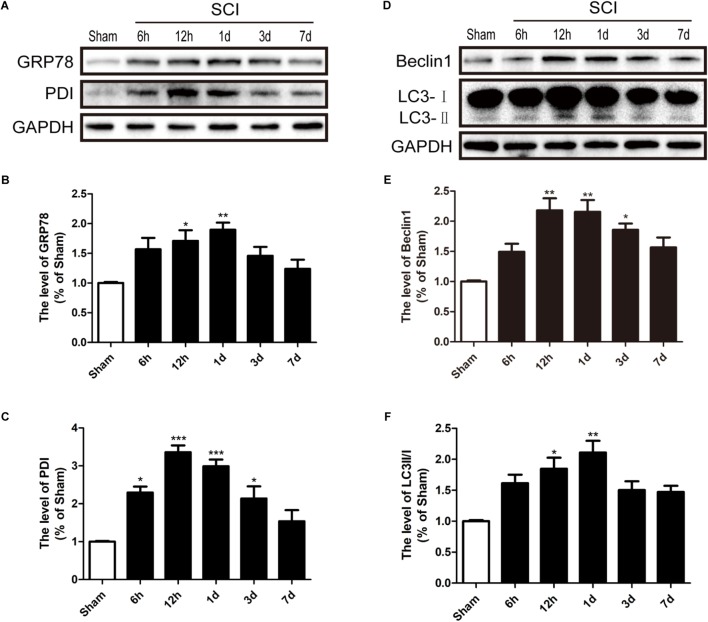
SCI activated ER stress and autophagy. **(A)** The expression levels of GRP78 and PDI in spinal cord from sham group and 6 h, 12 h, 1d, 3d, and 7d post-injury group. **(B,C)** Quantification of the western blotting data of GRP78 and PDI. **(D)** The expression Levels of Beclin1 and LC3B I/II in spinal cord at 6 h, 12 h, 1d, 3d, and 7d post-injury. **(E,F)** Quantification of the western blotting data of Beclin1 and LC3B I/II. The relative band density value was normalized to that of GAPDH. All data are presented as the mean ± SEM, *n* = 5. ^∗^*P* < 0.05, ^∗∗^*P* < 0.01, and ^∗∗∗^*P* < 0.001 versus the sham group.

To detect whether ER stress and autophagy were involved in SCI, we had detected the expression levels of ER stress makers (GRP78 and PDI) and autophagy makers (LC3 and Beclin-1) in a rat model of SCI. It was found that GRP78 and PDI levels were significantly up-regulated after SCI and peaked at 1d and 12 h post-surgery, respectively, (**Figures 1A–C**). Additionally, the LC3-II/I ratio and Beclin-1 levels were markedly increased 1d after SCI (**Figures 1D–F**). Based on the above findings, we concluded that SCI activated ER stress and autophagy, and 1 dpi rat model was used to perform the subsequent experiments.

### NaHS Administration Significantly Attenuates SCI-Induced ER Stress and Autophagy

**FIGURE 2:**
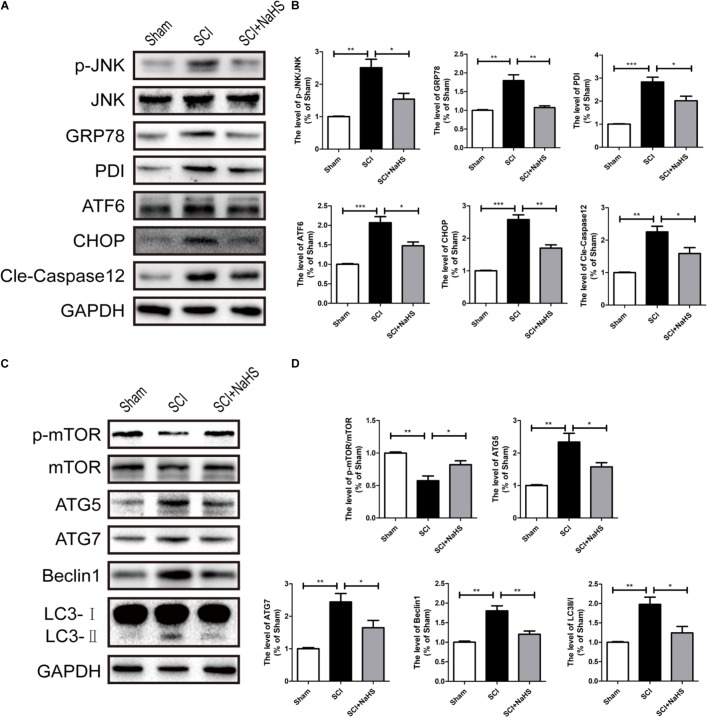
NaHS treatment significantly attenuated SCI-induced ER stress and autophagy at 1d after SCI. **(A)** Representative western blots of phosphor-JNK (p-JNK), JNK, GRP78, PDI, ATF6, CHOP, and Cle-caspase 12 in the sham, SCI model and SC+NaHS-treated groups. **(B)** Quantification of the western blotting data of p-JNK/JNK, GRP78, PDI, ATF6, CHOP, and Cle-caspase 12. **(C)** Representative western blots of phosphor-mTOR (p-mTOR), mTOR, ATG5, ATG7, Beclin1, and LC3B I/II for each group after SCI. **(D)** Quantification of the western blotting data of p-mTOR/mTOR, ATG5, ATG7, Beclin1, and LC3B I/II. The relative band density value was normalized to that of GAPDH. All data were presented as the mean ± SEM, *n* = 5. ^∗^*P* < 0.05, ^∗∗^*P* < 0.01 and ^∗∗∗^*P* < 0.001 versus the indicated group.

**FIGURE 3:**
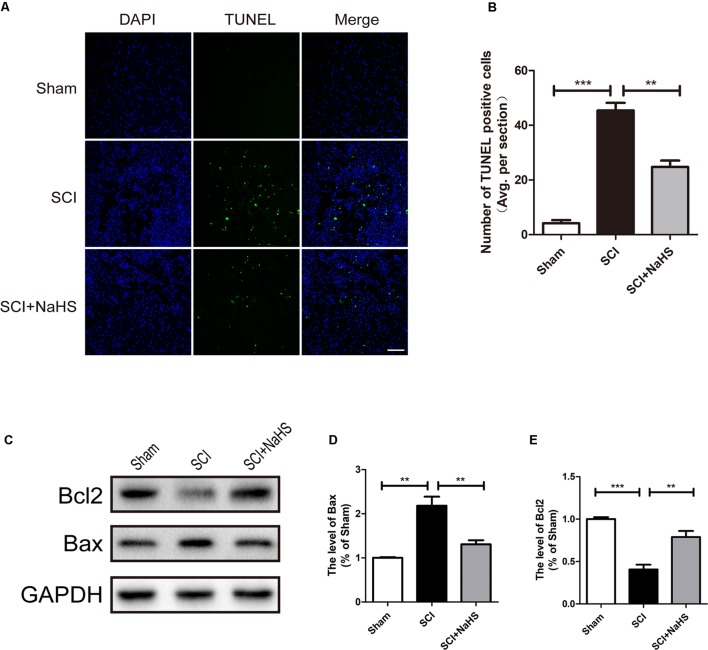
NaHS treatment blocked SCI-triggered apoptosis at 1d after SCI. **(A,B)** TUNEL staining in the sham, SCI model and SC+NaHS-treated groups, Scale bar = 50 μm. **(C–E)** Representative western blots of Bax and Bcl2 in the sham, SCI model and SC+NaHS-treated groups. All data were presented as the mean ± SEM, *n* = 3. ^∗∗^*P* < 0.01 and ^∗∗∗^*P* < 0.001 versus the indicated group.

Here, we try to evaluate whether NaHS administration could attenuate SCI-induced ER stress and autophagy. The rats were randomly divided into three groups: (i) sham group; (ii) SCI group; and (iii) SCI with NaHS treatment (SCI+NaHS) group. It was observed that the levels of GRP78, PDI, p-JNK, ATF6, CHOP, and Cle-Caspase 12 were significantly increased after SCI, and those increases were markedly inhibited by NaHS treatment (**Figures 2A,B**). Then, we had further detected the expression levels of p-mTOR, ATG5, ATG7, LC3, and Beclin-1. As shown in **Figures 2C,D**, the expression of ATG5, ATG7, LC3-II/I ratio, and Beclin-1 levels were increased after SCI, and NaHS administration dramatically attenuated the SCI-induced increases of ATG5, ATG7, LC3-II/I ratio, and Beclin-1 levels (**Figures 2C,D**). Moreover, NaHS treatment significantly blocked SCI-induced decrease of p-mTOR (**Figures 2C,D**). Additionally, using TUNEL staining, it was found that SCI dramatically triggered cell apoptosis(45.4 ± 2.81 versus 4.15 ± 1.18, ^∗∗∗^*P* < 0.001, *N* = 3) and NaHS treatment ameliorated it (45.4 ± 2.81 versus 24.78 ± 2.33, ^∗∗^*P* < 0.01, *N* = 3) (**Figures 3A,B**). Consistent with TUNEL, NaHS treatment significantly blocked SCI-induced decreases of Bcl-2 expression(0.79 ± 0.07 versus 0.41 ± 0.06, ^∗∗^*P* < 0.01, *N* = 3) and increases of Bax expression (1.31 ± 0.09 versus 2.2 ± 0.2, ^∗∗^*P* < 0.01, *N* = 3) (**Figures 3C–E**). Taken together, the above results indicate that NaHS treatment significantly attenuated the levels of ER stress and autophagy after SCI.

### SCI Induces the Loss of TJ and AJ Proteins

**FIGURE 4:**
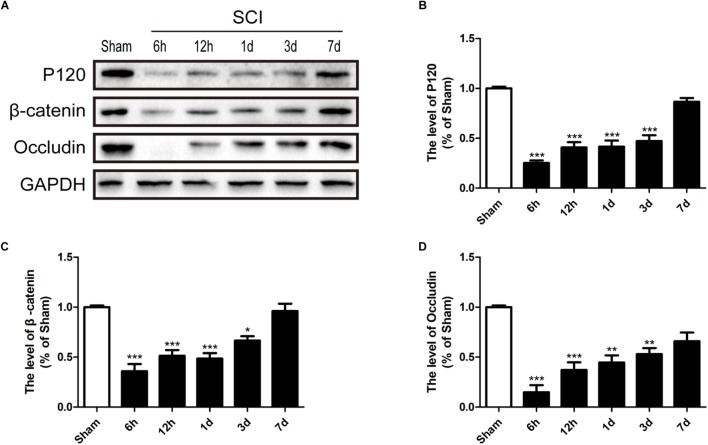
SCI induces the loss of TJ and AJ proteins. **(A)** Representative western blotting results for TJ proteins (Occludin) and AJ proteins (P120 and β-catenin) in the sham group, 6 h, 12 h, 1d, 3d, and 7d after SCI group. **(B–D)** Quantification of the expression of Occludin, P120, and β-catenin. The relative band density value was normalized to that of GAPDH. All data are presented as the mean ± SEM, *n* = 5. ^∗^*P* < 0.05, ^∗∗^*P* < 0.01, and ^∗∗∗^*P* < 0.001 versus the sham group.

It is well known that the TJs and AJs in the ECs of blood vessels is essential for maintenance of BSCB integrity (Bartanusz et al., 2011; Zheng et al., 2017). Thus, we had evaluated the expression levels of Occludin, P120 and β-catenin to determine whether levels of TJ (Occludin) and AJ (P120 and β-catenin) proteins were altered in response to SCI. Compared with sham group, Occludin, P120 and β-catenin were significantly decreased 6 h, 12 h, 1d, and 3d after SCI (**Figures 4A–D**). In conclusion, consistence with previous studies (Lee J.Y. et al., 2012), these data suggests that SCI induced the loss of TJ and AJ proteins.

### NaHS Administration Prevents the Loss of TJ and AJ Proteins by Inhibiting ER Stress and Autophagy After SCI

**FIGURE 5:**
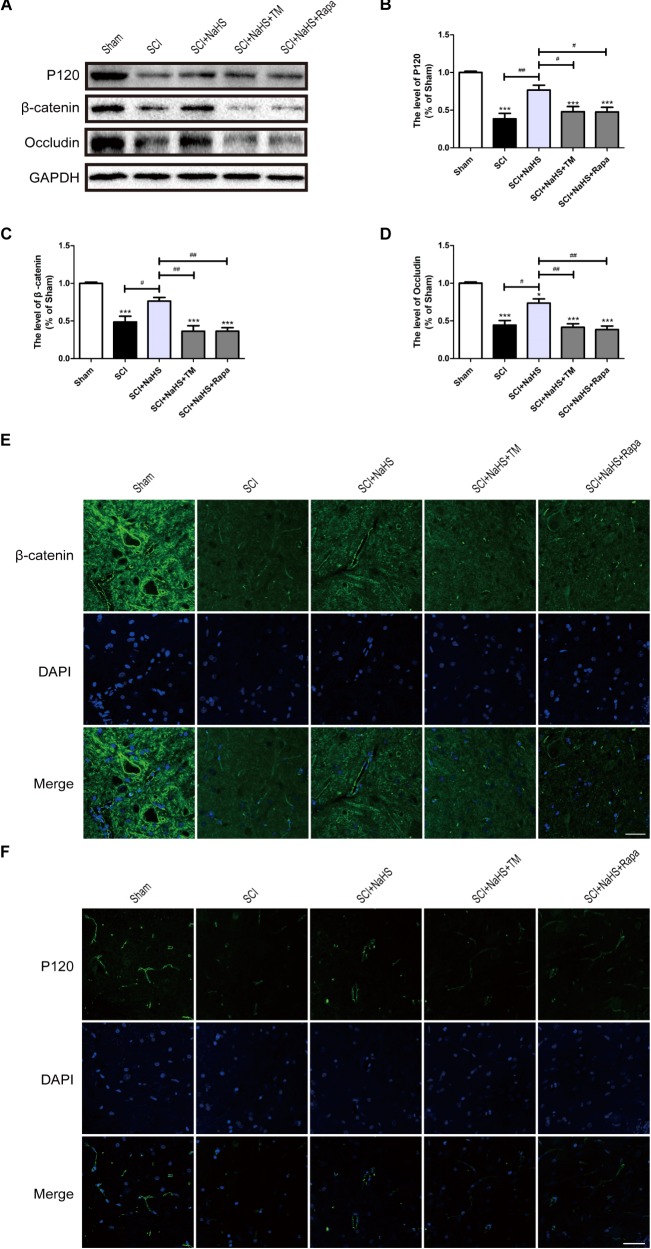
NaHS prevents the loss of TJ and AJ proteins by inhibiting ER stress and autophagy at 1d post-SCI. **(A)** TM and Rapa were applied to specifically activate ER stress and autophagy, respectively. The rats were randomly divided into five groups: (i) the Sham group; (ii) the SCI model group; (iii) the SCI model with NaHS treatment (SCI + NaHS) group; (iv) the SCI model with NaHS and TM treatment (SCI + NaHS + TM) group; and (v) the SCI model with NaHS and Rapa treatment (SCI + NaHS + Rapa) group. Representative western blots of P120, β-catenin and Occludin from each group. **(B–D)** Quantification of the expression of P120, β-catenin, and Occludin. The relative band density value was normalized to that of GAPDH. All data are presented as the mean ± SEM, *n* = 5. ^∗∗∗^*P* < 0.001 versus the sham group. ^#^*P* < 0.05 and ^##^*P* < 0.01 versus the indicated group. **(E,F)** Representative fluorescence images of β-catenin and P120 (Green) in sections from the spinal cord tissue in each group. Scale bar = 50 μm, *n* = 5.

To determine whether NaHS prevents the loss of TJ and AJ proteins after SCI, we examined the expression levels of TJ and AJ proteins after NaHS treatment for SCI. Western blotting results had showed that the levels of Occludin, P120 and β-catenin in spinal cord were decreased after SCI, which is remarkably blocked by NaHS treatment (**Figures 5A–D**). To further evaluate whether NaHS treatment prevents the loss of TJs and AJs after SCI by inhibiting ER stress and autophagy, tunicamycin (TM, a specific ER stress activator) and rapamycin (Rapa, a specific autophagy activator) were used to activate ER stress and autophagy, respectively. As shown in **Figures 5A–D**, both TM and Rapa treatment significantly reversed the protective effect of NaHS against SCI-induced loss of TJ and AJ proteins. Immunofluorescence staining results had further confirmed the effect of NaHS (**Figures 5E,F**). The above results demonstrates that NaHS treatment prevented the loss of TJs and AJs by inhibiting ER stress and autophagy.

### ER Stress Triggers Autophagy After SCI

**FIGURE 6:**
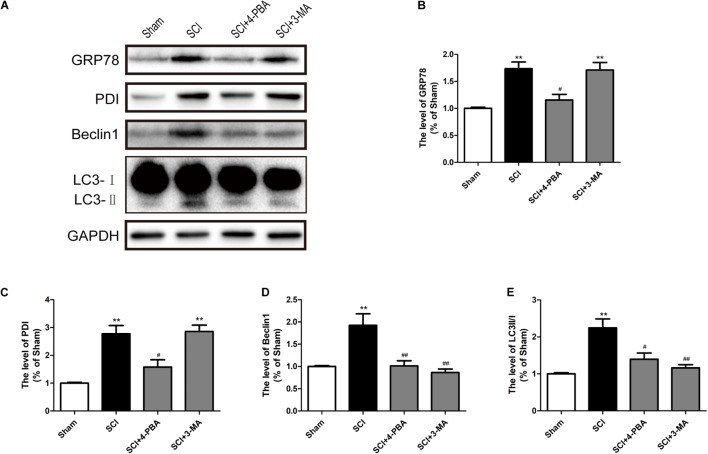
ER stress triggers autophagy after SCI. **(A)** 4-PBA and 3-MA were applied to specifically inhibit ER stress and autophagy. Representative western blots of GRP78, PDI, Beclin1, and LC3B I/II are shown. **(B–E)** Quantification of the expression of GRP78, PDI, Beclin1, and LC3B I/II. The relative band density value was normalized to that of GAPDH. All data are presented as the mean ± SEM, *n* = 5. ^∗∗^*P* < 0.01 versus the sham group, ^#^*P* < 0.05 and ^##^*P* < 0.01 versus the SCI group.

Prior reports have indicated that ER stress can mediate autophagy (Kouroku et al., 2007; Lee H. et al., 2012). To further confirm the connection between ER stress and autophagy during SCI, 4-PBA (a classical ER stress inhibitor) and 3-MA (a classical autophagy inhibitor) were applied to treatment for SCI. It was observed that 4-PBA not only suppressed ER stress, as indicated by the reduction of GRP78 and PDI, but also inhibited autophagy, as evidenced by the reduction of Beclin-1 and the LC3-II/I ratio (**Figures 6A–C**). However, 3-MA only inhibited the expression of Beclin-1 and the LC3-II/I ratio, without obvious effects on GRP78 and PDI (**Figures 6A,D,E**). The above results have suggested that SCI-induced ER stress significantly triggered autophagy.

### NaHS Administration Decreases BSCB Permeability and Improves Functional Recovery by Inhibiting ER Stress and Autophagy After SCI

**FIGURE 7:**
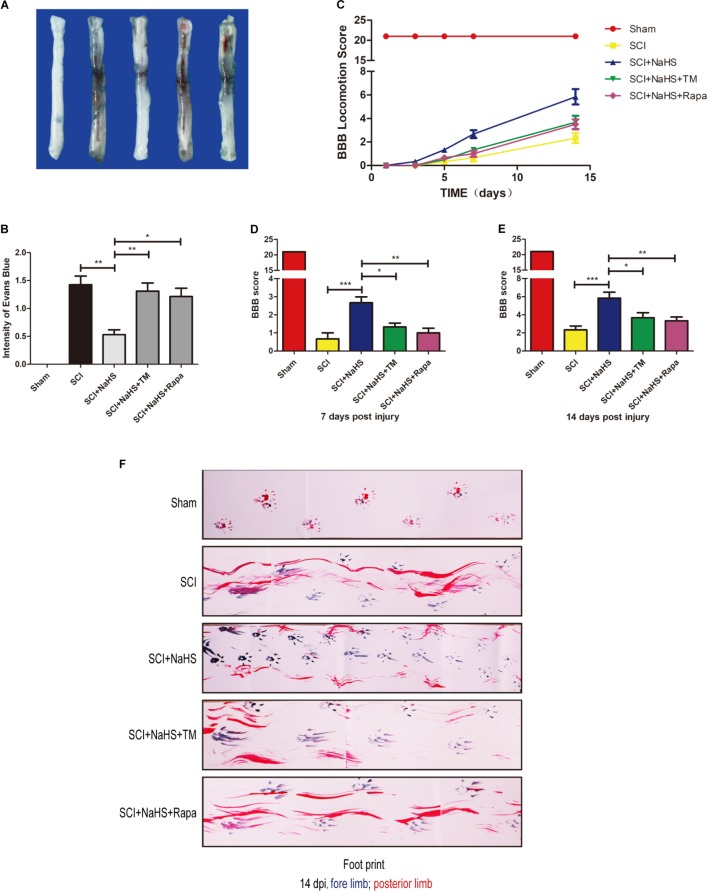
NaHS prevents the disruption of BSCB and improves the functional recovery by inhibiting ER stress and autophagy after SCI. **(A,B)** Representative whole spinal cord tissues and quantification of BSCB permeability for each group, indicating that EB dye permeabilized into the spinal cord. All data are presented as the mean ± SEM, *n* = 5. ^∗^*P* < 0.05 and ^∗∗^*P* < 0.01 versus the SCI + NaHS group. **(C)** The BBB locomotion scores of the different groups 1, 3, 5, 7, and 14d after SCI, *n* = 10. **(D,E)** Quantification of the BBB locomotion scores at 7 and 14d from **(C)**. All data are presented as the mean ± SEM, *n* = 10. ^∗^*P* < 0.05, ^∗∗^*P* < 0.01 and ^∗∗∗^*P* < 0.001 versus the SCI + NaHS group. **(F)** Footprint analysis results from the different groups.

To determine whether NaHS treatment mitigates SCI-induced increases of permeability after SCI, EB dye was used to examine the effect of NaHS on BSCB permeability at 1d post-SCI. As shown in **Figures 7A,B**, compared with the sham group, EB dye extravasation was significantly increased in SCI group, suggesting that the integrity of BSCB was disrupted after SCI. After administration of NaHS, the content of EB was markedly decreased compared with that in SCI group. However, both TM and Rapa significantly reduced the effect of NaHS on BSCB integrity(1.31 ± 0.15 versus 0.53 ± 0.09, ^∗∗^*P* < 0.01; 121 ± 0.15 versus 0.53 ± 0.09, ^∗^*P* < 0.05) (**Figures 7A,B**). We also assessed the BBB rating scale and footprint analysis to evaluate the locomotor recovery in rats after SCI. There were no remarkable differences in BBB scores among the SCI, SCI+NaHS, SCI+NaHS+TM, and SCI+NaHS+Rapa groups 1, 3, and 5d after injury. However, 7 and 14d after injury, it was observed that the BBB score of SCI+NaHS group was higher than the scores of the SCI(5.83 ± 0.65 versus 2.33 ± 0.42, ^∗∗∗^*P* < 0.001, *N* = 10), SCI+NaHS+TM (5.83 ± 0.65 versus 3.67 ± 0.56, ^∗^*P* < 0.05, *N* = 10), and SCI+NaHS+Rapa groups(5.83 ± 0.65 versus 3.33 ± 0.42, ^∗∗^*P* < 0.01, *N* = 10) (**Figures 7C–E**). In footprint analysis, NaHS-treated rats exhibited coordinated crawling of posterior limb (red ink) at 14d after SCI. However, the rats from SCI, SCI+NaHS+TM, and SCI+NaHS+Rapa groups still showed uncoordinated crawling and extensive dragging (**Figure 7F**, *N* = 10). These data indicates that NaHS treatment effectively prevented BSCB disruption and improved motor functional recovery by inhibiting ER stress and autophagy after SCI.

### NaHS Administration Inhibits ER Stress and Autophagy in OGD-Treated ECs

**FIGURE 8:**
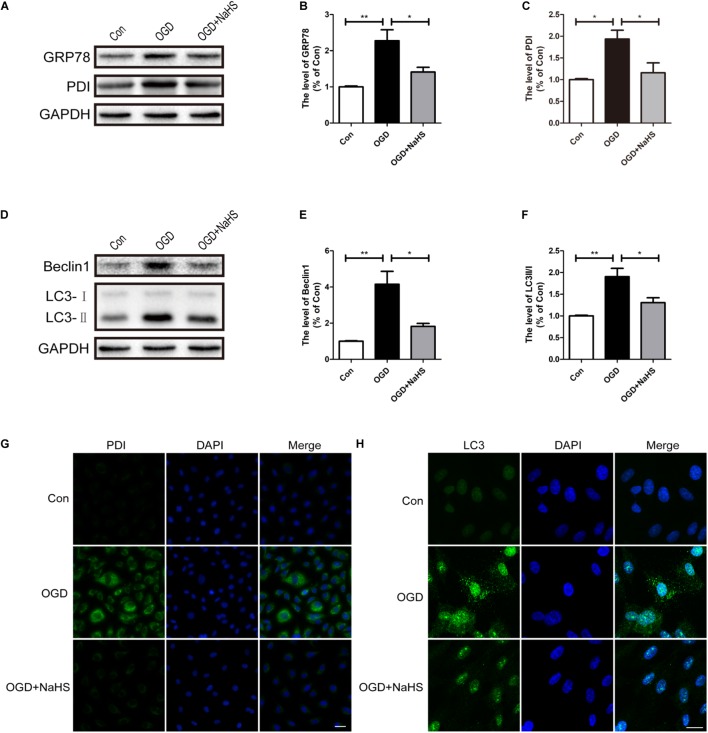
NaHS treatment inhibits the activation of ER stress and autophagy in OGD-treated HUVECs. **(A)** Representative western blots of GRP78 and PDI in HUVECs subjected to OGD and treated with NaHS. **(B,C)** Quantification of the expression of GRP78 and PDI. The relative band density value was normalized to that of GAPDH. All data are presented as the mean ± SEM. ^∗^*P* < 0.05 and ^∗∗^*P* < 0.01 versus the OGD group. **(D)** Representative western blots of Beclin1 and LC3B I/II for each group after OGD. **(E,F)** Quantification of the expression of Beclin1 and LC3B I/II. All data are presented as the mean ± SEM. ^∗^*P* < 0.05 and ^∗∗^*P* < 0.01 versus the OGD group. **(G,H)** Representative fluorescence images of PDI and LC3 (Green) in HUVECs. The nucleus is labeled with DAPI (blue). Scale bar = 50 μm (PDI), and scale bar = 25 μm (LC3), *n* = 3.

Here, we had further determined the effect of NaHS treatment on ER stress and autophagy *in vitro* using OGD treating HUVECs model. Compared with the control group, OGD treatment had up-regulated the expression levels of GRP78 and PDI, and these increases was significantly inhibited by NaHS administration (**Figures 8A–C**). In addition, consistence with the western blotting results, the OGD-treated group exhibited an increased fluorescence intensity of PDI. However, NaHS treatment markedly weakened the fluorescence intensity of PDI (**Figure 8G**). Furthermore, we had also detected the expression levels of the markers of autophagy. Compared with control group, the levels of Beclin-1 and LC3-II/I ratio were significantly increased in the OGD-treated group, which was dramatically blocked by NaHS administration (**Figures 8D–F**). Moreover, the immunofluorescence staining findings had also showed that the OGD group exhibited an enhanced fluorescence intensity of LC3 when compared with that of the control group, which was significantly decreased after treatment with NaHS (**Figure 8H**). Taken Together, the above findings indicate that NaHS treatment significantly inhibited ER stress and autophagy *in vitro*.

### NaHS Administration Attenuates the Loss of TJ and AJ Proteins by Inhibiting ER Stress and Autophagy *in vitro*

**FIGURE 9:**
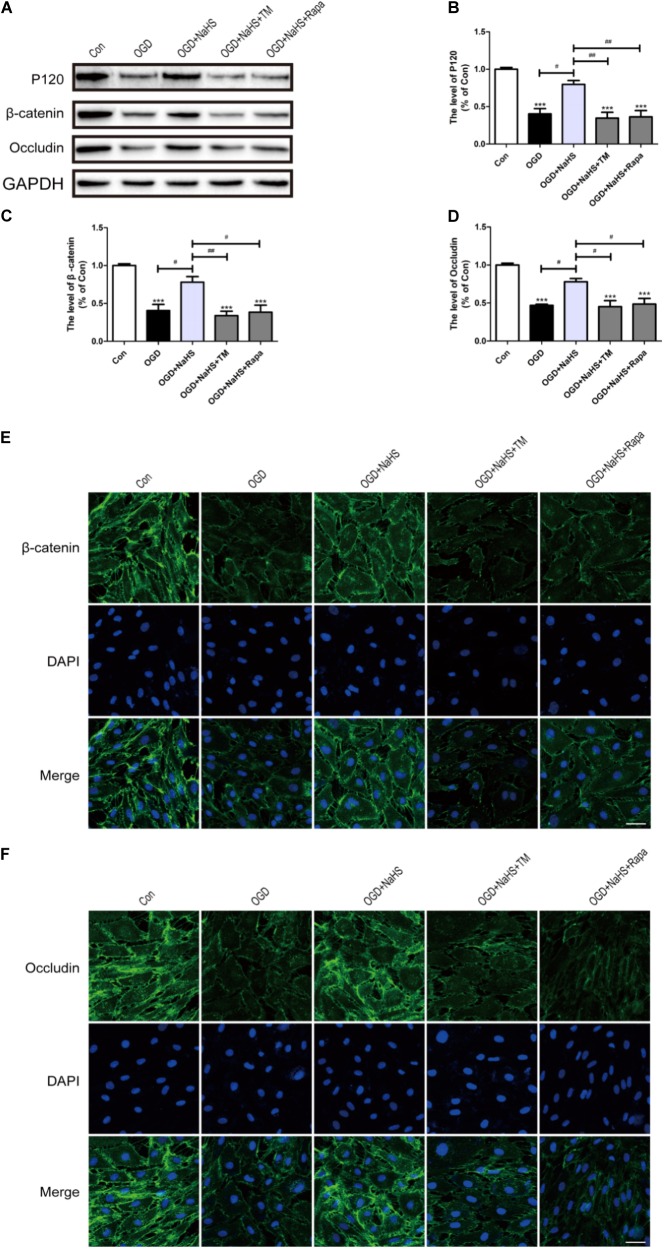
NaHS protects OGD-treated HUVECs by inhibiting ER stress and autophagy *in vitro*. **(A)** Representative western blots of P120, β-catenin and Occludin for each group. **(B–D)** Quantification of the expression of P120, β-catenin and Occludin. The relative band density value was normalized to that of GAPDH. All data are presented as the mean ± SEM. ^∗∗∗^*P* < 0.001 versus the Con group. ^#^*P* < 0.05 and ^##^*P* < 0.01 versus the indicated group. **(E,F)** Representative fluorescence images of β-catenin and Occludin (Green) in HUVECs for each group. The nucleus is labeled with DAPI (blue). Scale bar = 50 μm, *n* = 3.

To further determine whether the protective effect of NaHS on BSCB is related to the inhibition of ER stress and autophagy, NaHS, TM, and Rapa were administered to HUVECs. Consistent with the findings obtained *in vivo*, the expression levels of P120, β-catenin and Occludin were decreased in the OGD group and significantly up-regulated in the OGD+NaHS group. However, both TM and Rapa markedly weakened the protective effect of NaHS (**Figures 9A–D**). In addition, immunofluorescence staining results had also showed that the intensity of β-catenin and Occludin in the OGD group was decreased compared to that of the control group. However, NaHS treatment significantly reversed the destructive effect of OGD, as evidenced by the increased intensity of β-catenin and Occludin (**Figures 9E,F**). Both TM and Rapa abolished such effect of NaHS. These data indicates that NaHS effectively attenuated the loss of TJs and AJs by inhibiting ER stress and autophagy in OGD-treated ECs.

## Discussion

Spinal cord injury is a devastating neurological disease that affects thousands of patients every year. A series of secondary injuries, including vascular changes, oxidative stress, and apoptosis, are considered as the major causes of disability (Fan et al., 2013; Colon et al., 2016; Izmailov et al., 2017). Thus, it is important to explore the effective therapeutic treatments for SCI and reduce SCI-induced tissue damage and neurological disorders. In present study, we had demonstrated that NaHS administration effectively decreases the degradation of TJ and AJ proteins, prevents BSCB permeability, and ultimately protects the spinal cord against secondary injury to improve functional recovery. The inhibitions of ER stress and autophagy were the potential molecular mechanisms underlying NaHS treatment for SCI.

As a H_2_S donor, NaHS has been widely used to assess the therapeutic potential of exogenous H_2_S delivery. Previous study employed aqueous NaHS solutions to assess the rat aortic ring response *in vitro* (Zhao et al., 2001). NaHS mitigated the degree of acute lung injury by attenuating IL-6 and IL-8 levels, while increasing IL-10 levels in the lung tissue and plasma (Li et al., 2008). Moreover, NaHS exhibited efficacy in preventing cell damage caused by ROS in brain cells (Whiteman et al., 2005). Recent studies had also indicated that NaHS has a protective effect in peripheral nerve injury and spinal cord ischemia reperfusion (Park et al., 2015; Xie et al., 2017b). Our findings had indicated that NaHS could improve functional recovery after SCI, suggesting that NaHS probably represents a potential therapeutic approach for traumatic SCI.

It is well known that the maintenance of BSCB integrity effectively promotes central nervous system (CNS) recovery after SCI, and TJ and AJ proteins are the crucial components of BSCB (Lee J.Y. et al., 2012). The disruption of BSCB allows neutrophils and other immune cells to infiltrate the area of injury, resulting in serious secondary injuries (Penas et al., 2007). Therefore, regulation of TJ and AJ proteins may be the potential mechanism of NaHS-induced neuroprotective role during SCI. In addition, it has been demonstrated that the administration of NaHS protects the blood-brain barrier (BBB) integrity after cerebral ischemia and traumatic brain injury (Wang et al., 2014; Xu et al., 2018). The BSCB is analogous to the BBB in that it is selectively permeable and blocks the entry of blood cells and other molecules into the CNS (Bartanusz et al., 2011). Thus, we evaluated the effect of NaHS on the BSCB after SCI. We discovered that NaHS treatment reduced the leakage of EB dye. In addition, the decreases of TJ (P120, β-catenin) and AJ (Occludin) proteins were significantly inhibited by NaHS treatment when comparing with the permeability of the BSCB during SCI. Furthermore, NaHS treatment inhibited the degradation of TJ and AJ proteins under OGD conditions *in vitro*. Taken together, these findings indicated that NaHS treatment promotes to maintain BSCB integrity following SCI.

ER stress occurs when misfolded proteins accumulate in the ER lumen and cause ER dysfunction, which is known as unfolded protein response (UPR) (Schroder and Kaufman, 2005). ER stress is one of the crucial molecular mechanisms underlies the pathogenesis of SCI. Recent studies indicated that the inhibition of ER stress prevents the disruption of the BSCB (Zheng et al., 2017), and NaHS treatment could suppress the activation of ER stress (Xu et al., 2017). Consistent with the prior study, we had evaluated the role of ER stress in the effect of NaHS on BSCB restoration after SCI or OGD, and found that NaHS treatment inhibited the activation of ER stress. Furthermore, the activation of ER stress with TM significantly reversed the protective effect of NaHS on BSCB restoration and consequently functional motor recovery after SCI. These data collectively suggest that NaHS treatment rescue the BSCB disruption from SCI via inhibiting ER stress.

Autophagy, a catabolic cellular organelle process, removes unwanted cellular components through double-membrane autophagosomes fused with lysosomes, and, thus, is essential for survival, differentiation, development and homeostasis (Noda and Inagaki, 2015). Previous studies had demonstrated that autophagy exerts a destructive role during SCI and inhibiting autophagy can promote locomotor functional recovery of mice (Wu et al., 2018). Consistent with prior study, our findings had revealed that autophagy is markedly activated during the acute phase of SCI. NaHS administration downregulated the expression of Beclin1 and the LC3-II/I ratio *in vivo* and *in vitro*, and prevented the loss of TJs and AJs protein. However, the role of autophagy in SCI and the reciprocal regulation between H_2_S and autophagy remained controversial. Recent study had reported that H_2_S promotes autophagy via the PI3K/Akt/mTOR signaling pathway in hepatocellular carcinoma cells (Wang et al., 2017). Moreover, autophagy stimulation or enhancement can improve neuron protection and functional recovery during SCI (Bai et al., 2017; Hu et al., 2017). It is well known that the pathological status of spinal cord is changing after SCI. We speculate that the different periods of SCI maybe the caused factor for the different role of autophagy and H_2_S on SCI, especially in acute phase and chronic phase.

Our mechanism studies have shown that ER stress and autophagy activation are both involved in NaHS treatment for BSCB disruption during SCI. However, whether ER stress has a cross-talking with autophagy during NaHS treatment for BSCB is still unclear. Prior and our current study has demonstrated that there are mutual cross-talking between ER stress and autophagy (Hoyer-Hansen and Jaattela, 2007). Additionally, some studies have indicated that activated ER stress can trigger autophagy (Chandrika et al., 2015; Feng et al., 2017). Furthermore, ER stress can induce the initiation of autophagosome formation via the IRE1/JNK signaling pathway (Ogata et al., 2006) and PERK/eIF2α signaling pathway (Kouroku et al., 2007; B’Chir et al., 2013). In our present study, 4-PBA markedly blocked SCI-induced autophagy, however, the autophagy inhibitor (3-MA) can only inhibit autophagy activity without remarkable effects on ER stress. These results suggests that NaHS treatment blocked SCI-induced ER stress and ER stress-associated autophagy, subsequently ameliorated SCI.

An interesting finding in present study was verified that inhibition of autophagy and ER stress contributes to the H_2_S treatment for SCI. It has been demonstrated that H_2_S regulates oxidative stress (Kimura et al., 2010; Xie et al., 2017b). But the role of H_2_S on oxidative stress remained controversial. Some studies demonstrate that H_2_S directly suppress oxidative stress (Kimura et al., 2010), whereas the others studies have demonstrate H2S has a deleterious effect on oxidative stress via increasing the formation of reactive oxygen species and leading to glutathione (GSH) depletion (Truong, 2006). The present study did not further provide the evidence of the relationship between H2S and oxidative stress in the disruption of BSCB after SCI. Some studies has reported that concentrations of H_2_S is the conclusive factor for its significant physiological and toxicological roles. It has demonstrated that the concentrations (∼15 mM) of H_2_S quickly causes death (Roth, 1993). However, levels of H_2_S < 0.1 mM are generated endogenously and have been shown to affect neuronal communication and regulation of smooth muscle tone(Wang, 2002; Teague et al., 2010). Thus, it is important to utilize the reasonable concentration of H_2_S for treatment the related disease. In our current study, NaHS (5.6 mg/kg) and NaHS (100 μM) was used *in vivo* and *in vitro*, which is less than 0.1 mM. Therefore, it is reasonable to speculate that the NaHS treatment can inhibit oxidative stress and ameliorate the disruption of BSCB after SCI in our study.

In summary, our study has demonstrated that NaHS administration significantly ameliorates the disruption of BSCB and subsequently improves functional motor recovery after SCI. Inhibition of ER stress and ER stress-associated autophagy were involved in NaHS treatment for SCI. Our finding suggested that rational H_2_S treatment promotes the recovery of SCI, which is the potential therapeutic strategies for SCI.

## Author Contributions

JX and HX conceived and designed the experiments. HW and WH performed the experiments. HW and YW performed statistical analysis and wrote the paper. JL, KX, ZL, QW, YL, LX, JW, and HH provided assistance with experiments. All authors discussed the results and approved the final manuscript.

## Conflict of Interest Statement

The authors declare that the research was conducted in the absence of any commercial or financial relationships that could be construed as a potential conflict of interest.

## References

[B1] Abe K. Kimura H. (1996). The possible role of hydrogen sulfide as an endogenous neuromodulator. *J. Neurosci.* 16 1066–1071. 10.1523/JNEUROSCI.16-03-01066.1996 8558235 PMC6578817

[B2] Acioglu C. Mirabelli E. Baykal A. T. Ni L. Ratnayake A. Heary R. F. (2016). Toll like receptor 9 antagonism modulates spinal cord neuronal function and survival: Direct versus astrocyte-mediated mechanisms. *Brain Behav. Immun.* 56 310–324. 10.1016/j.bbi.2016.03.027 27044334

[B3] Bai L. Mei X. Wang Y. Yuan Y. Bi Y. Li G. (2017). The Role of Netrin-1 in Improving Functional Recovery through Autophagy Stimulation Following Spinal Cord Injury in Rats. *Front Cell Neurosci* 11 350. 10.3389/fncel.2017.00350 29209172 PMC5701630

[B4] Bartanusz V. Jezova D. Alajajian B. Digicaylioglu M. (2011). The blood-spinal cord barrier: morphology and clinical implications. *Ann. Neurol.* 70 194–206. 10.1002/ana.22421 21674586

[B5] B’Chir W. Maurin A. C. Carraro V. Averous J. Jousse C. Muranishi Y. (2013). The eIF2alpha/ATF4 pathway is essential for stress-induced autophagy gene expression. *Nucleic Acids Res.* 41(16), 7683–7699. 10.1093/nar/gkt563 23804767 PMC3763548

[B6] Calvert J. W. Jha S. Gundewar S. Elrod J. W. Ramachandran A. Pattillo C. B. (2009). Hydrogen sulfide mediates cardioprotection through Nrf2 signaling. *Circ. Res.* 105 365–374. 10.1161/CIRCRESAHA.109.199919 19608979 PMC2735849

[B7] Chandrika B. B. Yang C. Ou Y. Feng X. Muhoza D. Holmes A. F. (2015). Endoplasmic Reticulum Stress-Induced Autophagy Provides Cytoprotection from Chemical Hypoxia and Oxidant Injury and Ameliorates Renal Ischemia-Reperfusion Injury. *PLoS ONE* 10(10), e0140025. 10.1371/journal.pone.0140025 26444017 PMC4596863

[B8] Chen J. Gao J. Sun W. Li L. Wang Y. Bai S. (2016). Involvement of exogenous H2S in recovery of cardioprotection from ischemic post-conditioning via increase of autophagy in the aged hearts. *Int. J. Cardiol.* 220 681–692. 10.1016/j.ijcard.2016.06.200 27393850

[B9] Colon J. M. Torrado A. I. Cajigas A. Santiago J. M. Salgado I. K. Arroyo Y. (2016). Tamoxifen Administration Immediately or 24 Hours after Spinal Cord Injury Improves Locomotor Recovery and Reduces Secondary Damage in Female Rats. *J. Neurotrauma* 33(18), 1696–1708. 10.1089/neu.2015.4111 26896212 PMC5035917

[B10] Ding W. X. Yin X. M. (2008). Sorting, recognition and activation of the misfolded protein degradation pathways through macroautophagy and the proteasome. *Autophagy* 4 141–150. 10.4161/auto.5190 17986870

[B11] Fan Z. K. Cao Y. Lv G. Wang Y. S. Guo Z. P. (2013). The effect of cigarette smoke exposure on spinal cord injury in rats. *J. Neurotrauma* 30 473–479. 10.1089/neu.2012.2574 23234244 PMC3696935

[B12] Fang B. Li X. Q. Bi B. Tan W. F. Liu G. Zhang Y. (2015). Dexmedetomidine attenuates blood-spinal cord barrier disruption induced by spinal cord ischemia reperfusion injury in rats. *Cell Physiol. Biochem* 36 373–383. 10.1159/000430107 25967975

[B13] Feng D. Wang B. Wang L. Abraham N. Tao K. Huang L. (2017). Pre-ischemia melatonin treatment alleviated acute neuronal injury after ischemic stroke by inhibiting endoplasmic reticulum stress-dependent autophagy via PERK and IRE1 signalings. *J. Pineal Res.* 62. 10.1111/jpi.12395 28178380

[B14] Fiorucci S. Distrutti E. Cirino G. Wallace J. L. (2006). The emerging roles of hydrogen sulfide in the gastrointestinal tract and liver. *Gastroenterology* 131 259–271. 10.1053/j.gastro.2006.02.033 16831608

[B15] Gong L. Tang Y. An R. Lin M. Chen L. Du J. (2017). RTN1-C mediates cerebral ischemia/reperfusion injury via ER stress and mitochondria-associated apoptosis pathways. *Cell Death Dis* 8(10), e3080. 10.1038/cddis.2017.465 28981095 PMC5680587

[B16] He M. Ding Y. Chu C. Tang J. Xiao Q. Luo Z. G. (2016). Autophagy induction stabilizes microtubules and promotes axon regeneration after spinal cord injury. *Proc. Natl. Acad. Sci. U.S.A.* 113(40), 11324–11329. 10.1073/pnas.1611282113 27638205 PMC5056063

[B17] Hoyer-Hansen M. Jaattela M. (2007). Connecting endoplasmic reticulum stress to autophagy by unfolded protein response and calcium. *Cell Death. Differ* 14 1576–1582. 10.1038/sj.cdd.4402200 17612585

[B18] Hu J. Han H. Cao P. Yu W. Yang C. Gao Y. (2017). Resveratrol improves neuron protection and functional recovery through enhancement of autophagy after spinal cord injury in mice. *Am J Transl Res* 9 4607–4616. 29118921 PMC5666068

[B19] Izmailov A. A. Povysheva T. V. Bashirov F. V. Sokolov M. E. Fadeev F. O. Garifulin R. R. (2017). Spinal Cord Molecular and Cellular Changes Induced by Adenoviral Vector- and Cell-Mediated Triple Gene Therapy after Severe Contusion. *Front Pharmacol* 8 813. 10.3389/fphar.2017.00813 29180963 PMC5693893

[B20] Kamat P. K. Kyles P. Kalani A. Tyagi N. (2016). Hydrogen Sulfide Ameliorates Homocysteine-Induced Alzheimer’s Disease-Like Pathology, Blood-Brain Barrier Disruption, and Synaptic Disorder. *Mol. Neurobiol.* 53 2451–2467. 10.1007/s12035-015-9212-4 26019015 PMC4662933

[B21] Kimura Y. Goto Y. Kimura H. (2010). Hydrogen sulfide increases glutathione production and suppresses oxidative stress in mitochondria. *Antioxidants & Redox Signalling* 12 1. 10.1089/ars.2008.2282 19852698

[B22] Kouroku Y. Fujita E. Tanida I. Ueno T. Isoai A. Kumagai H. (2007). ER stress (PERK/eIF2alpha phosphorylation) mediates the polyglutamine-induced LC3 conversion, an essential step for autophagy formation. *Cell Death. Differ* 14 230–239. 10.1038/sj.cdd.4401984 16794605

[B23] Kuang X. Hu W. Yan M. Wong P. K. (2010). Phenylbutyric acid suppresses protein accumulation-mediated ER stress in retrovirus-infected astrocytes and delays onset of paralysis in infected mice. *Neurochem. Int.* 57 738–748. 10.1016/j.neuint.2010.08.010 20813146 PMC3402222

[B24] Lai E. Teodoro T. Volchuk A. (2007). Endoplasmic reticulum stress: signaling the unfolded protein response. *Physiology (Bethesda)* 22 193–201. 10.1152/physiol.00050.2006 17557940

[B25] Lee H. Noh J. Y. Oh Y. Kim Y. Chang J. W. Chung C. W. (2012). IRE1 plays an essential role in ER stress-mediated aggregation of mutant huntingtin via the inhibition of autophagy flux. *Hum. Mol. Genet.* 21 101–114. 10.1093/hmg/ddr445 21954231

[B26] Lee J. Y. Choi H. Y. Park C. S. Ju B. G. Yune T. Y. (2018). Mithramycin A Improves Functional Recovery by Inhibiting BSCB Disruption and Hemorrhage after Spinal Cord Injury. *J. Neurotrauma* 35 508–520. 10.1089/neu.2017.5235 29048243

[B27] Lee J. Y. Kim H. S. Choi H. Y. Oh T. H. Yune T. Y. (2012). Fluoxetine inhibits matrix metalloprotease activation and prevents disruption of blood-spinal cord barrier after spinal cord injury. *Brain* 135(Pt 8), 2375–2389. 10.1093/brain/aws171 22798270

[B28] Li J. Wang Q. Wang H. Wu Y. Yin J. Chen J. (2018). Lentivirus Mediating FGF13 Enhances Axon Regeneration after Spinal Cord Injury by Stabilizing Microtubule and Improving Mitochondrial Function. *J. Neurotrauma* 35 548–559. 10.1089/neu.2017.5205 28922963

[B29] Li T. Zhao B. Wang C. Wang H. Liu Z. Li W. (2008). Regulatory effects of hydrogen sulfide on IL-6, IL-8 and IL-10 levels in the plasma and pulmonary tissue of rats with acute lung injury. *Exp Biol Med (Maywood)* 233 1081–1087. 10.3181/0712-RM-354 18535161

[B30] Li X. Q. Chen F. S. Tan W. F. Fang B. Zhang Z. L. Ma H. (2017). Elevated microRNA-129-5p level ameliorates neuroinflammation and blood-spinal cord barrier damage after ischemia-reperfusion by inhibiting HMGB1 and the TLR3-cytokine pathway. *J Neuroinflammation* 14 205. 10.1186/s12974-017-0977-4 29061187 PMC5654055

[B31] Lin F. Liao C. Sun Y. Zhang J. Lu W. Bai Y. (2017). Hydrogen Sulfide Inhibits Cigarette Smoke-Induced Endoplasmic Reticulum Stress and Apoptosis in Bronchial Epithelial Cells. *Front Pharmacol* 8 675. 10.3389/fphar.2017.00675 29033840 PMC5625329

[B32] Magierowski M. Magierowska K. Hubalewska-Mazgaj M. Surmiak M. Sliwowski Z. Wierdak M. (2017). Cross-talk between hydrogen sulfide and carbon monoxide in the mechanism of experimental gastric ulcers healing, regulation of gastric blood flow and accompanying inflammation. *Biochem Pharmacol.* 10.1016/j.bcp.2017.11.020 29203367

[B33] Martelli A. Testai L. Breschi M.C. Blandizzi C. Virdis A. Taddei S. (2012). Hydrogen sulphide: novel opportunity for drug discovery. Med Res Rev 32 1093–1130. 10.1002/med.20234 23059761

[B34] Mecha M. Torrao A. S. Mestre L. Carrillo-Salinas F. J. Mechoulam R. Guaza C. (2012). Cannabidiol protects oligodendrocyte progenitor cells from inflammation-induced apoptosis by attenuating endoplasmic reticulum stress. *Cell Death Dis* 3 e331. 10.1038/cddis.2012.71 22739983 PMC3388241

[B35] Mercado G. Castillo V. Soto P. Lopez N. Axten J. M. Sardi S. P. (2018). Targeting PERK signaling with the small molecule GSK2606414 prevents neurodegeneration in a model of Parkinson’s disease. *Neurobiol. Dis.* 112 136–148. 10.1016/j.nbd.2018.01.004 29355603

[B36] Mizushima N. Komatsu M. (2011). Autophagy: renovation of cells and tissues. *Cell* 147 728–741. 10.1016/j.cell.2011.10.026 22078875

[B37] Noda N. N. Inagaki F. (2015). Mechanisms of Autophagy. *Annu Rev Biophys* 44 101–122. 10.1146/annurev-biophys-060414-034248 25747593

[B38] Ogata M. Hino S. Saito A. Morikawa K. Kondo S. Kanemoto S. (2006). Autophagy is activated for cell survival after endoplasmic reticulum stress. *Mol. Cell. Biol.* 26(24), 9220–9231. 10.1128/MCB.01453-06 17030611 PMC1698520

[B39] Park B. S. Kim H. W. Rhyu I. J. Park C. Yeo S. G. Huh Y. (2015). Hydrogen sulfide is essential for Schwann cell responses to peripheral nerve injury. *J. Neurochem.* 132 230–242. 10.1111/jnc.12932 25123509

[B40] Penas C. Guzman M. S. Verdu E. Fores J. Navarro X. Casas C. (2007). Spinal cord injury induces endoplasmic reticulum stress with different cell-type dependent response. *J. Neurochem.* 102 1242–1255. 10.1111/j.1471-4159.2007.04671.x 17578450

[B41] Roth S. H. (1993). Hydrogen Sulfide. *Handbook of Hazardous Materials*, 367–376. 10.1016/B978-0-12-189410-8.50036-5

[B42] Rzymski T. Milani M. Pike L. Buffa F. Mellor H. R. Winchester L. (2010). Regulation of autophagy by ATF4 in response to severe hypoxia. *Oncogene* 29(31), 4424–4435. 10.1038/onc.2010.191 20514020

[B43] Schroder M. Kaufman R. J. (2005). The mammalian unfolded protein response. *Annu. Rev. Biochem.* 74 739–789 10.1146/annurev.biochem.73.011303.074134 15952902

[B44] Teague B. Asiedu S. Moore P. K. (2010). The smooth muscle relaxant effect of hydrogen sulphide in vitro: evidence for a physiological role to control intestinal contractility. *Br. J. Pharmacol.* 137 139–145. 10.1038/sj.bjp.0704858 12208769 PMC1573483

[B45] Truong D. H. Eghbal M. A. Hindmarsh W. Roth S. H. O’Brien P. J. (2006). Molecular mechanisms of hydrogen sulfide toxicity. *Drug Metab. Rev* 38 733–744. 10.1080/03602530600959607 17145698

[B46] Wang R. (2002). Two’s company, three’s a crowd: can H2S be the third endogenous gaseous transmitter? Faseb Journal Official Publication of the Federation of American Societies for Experimental Biology 16 1792. 10.1096/fj.02-0211hyp 12409322

[B47] Wang S. S. Chen Y. H. Chen N. Wang L. J. Chen D. X. Weng H. L. (2017). Hydrogen sulfide promotes autophagy of hepatocellular carcinoma cells through the PI3K/Akt/mTOR signaling pathway. *Cell Death Dis* 8 e2688. 10.1038/cddis.2017.18 28333142 PMC5386547

[B48] Wang Y. Jia J. Ao G. Hu L. Liu H. Xiao Y. (2014). Hydrogen sulfide protects blood-brain barrier integrity following cerebral ischemia. *J. Neurochem.* 129 827–838. 10.1111/jnc.12695 24673410

[B49] Whiteman M. Cheung N. S. Zhu Y. Z. Chu S. H. Siau J. L. Wong B. S. (2005). Hydrogen sulphide: a novel inhibitor of hypochlorous acid-mediated oxidative damage in the brain? Biochem Biophys Res Commun 326 794–798. 10.1016/j.bbrc.2004.11.110 15607739

[B50] Wu F. Wei X. Wu Y. Kong X. Hu A. Tong S. (2018). Chloroquine promotes the recovery of acute spinal cord injury by inhibiting autophagy-associated inflammation and endoplasmic reticulum stress. *J Neurotrauma.* 10.1089/neu.2017.5414 29316847

[B51] Wu J. Zhao Z. Kumar A. Lipinski M. M. Loane D. J. Stoica B. A. (2016). Endoplasmic Reticulum Stress and Disrupted Neurogenesis in the Brain Are Associated with Cognitive Impairment and Depressive-Like Behavior after Spinal Cord Injury. *J. Neurotrauma* 33(21), 1919–1935. 10.1089/neu.2015.4348 27050417 PMC5105355

[B52] Xiao J. Zhu X. Kang B. Xu J. Wu L. Hong J. (2015). Hydrogen Sulfide Attenuates Myocardial Hypoxia-Reoxygenation Injury by Inhibiting Autophagy via mTOR Activation. *Cell Physiol. Biochem* 37 2444–2453. 10.1159/000438597 26658637

[B53] Xiao T. Luo J. Wu Z. Li F. Zeng O. Yang J. (2016). Effects of hydrogen sulfide on myocardial fibrosis and PI3K/AKT1-regulated autophagy in diabetic rats. *Mol Med Rep* 13 1765–1773. 10.3892/mmr.2015.4689 26676365

[B54] Xie L. Yu S. Wang Z. Yang K. Liu Z. Li C. (2017a). Nicotinamide Adenine Dinucleotide Protects against Spinal Cord Ischemia Reperfusion Injury-Induced Apoptosis by Blocking Autophagy. *Oxid Med Cell Longev* 2017 7063874. 10.1155/2017/7063874 28367271 PMC5359458

[B55] Xie L. Yu S. Yang K. Li C. Liang Y. (2017b). Hydrogen Sulfide Inhibits Autophagic Neuronal Cell Death by Reducing Oxidative Stress in Spinal Cord Ischemia Reperfusion Injury. *Oxid Med Cell Longev* 2017: 8640284. 10.1155/2017/8640284 28685010 PMC5480044

[B56] Xu D. Jin H. Wen J. Chen J. Chen D. Cai N. (2017). Hydrogen sulfide protects against endoplasmic reticulum stress and mitochondrial injury in nucleus pulposus cells and ameliorates intervertebral disc degeneration. *Pharmacol. Res.* 117 357–369. 10.1016/j.phrs.2017.01.005 28087442

[B57] Xu K. Wu F. Xu K. Li Z. Wei X. Lu Q. (2018). NaHS restores mitochondrial function and inhibits autophagy by activating the PI3K/Akt/mTOR signalling pathway to improve functional recovery after traumatic brain injury. *Chemico-Biological Interactions.* 10.1016/j.cbi.2018.02.028 29567101

[B58] Yang Z. Klionsky D. J. (2010). Eaten alive: a history of macroautophagy. *Nat. Cell Biol.* 12 814–822. 10.1038/ncb0910-814 20811353 PMC3616322

[B59] Yu Q. Wang B. Zhao T. Zhang X. Tao L. Shi J. (2017). NaHS Protects against the Impairments Induced by Oxygen-Glucose Deprivation in Different Ages of Primary Hippocampal Neurons. *Front Cell Neurosci* 11 67. 10.3389/fncel.2017.00067 28326019 PMC5339257

[B60] Zhao W. Zhang J. Lu Y. Wang R. (2001). The vasorelaxant effect of H(2)S as a novel endogenous gaseous K(ATP) channel opener. *EMBO J.* 20(21), 6008–6016. 10.1093/emboj/20.21.6008 11689441 PMC125693

[B61] Zheng B. Ye L. Zhou Y. Zhu S. Wang Q. Shi H. (2016). Epidermal growth factor attenuates blood-spinal cord barrier disruption via PI3K/Akt/Rac1 pathway after acute spinal cord injury. *J. Cell Mol. Med.* 20 1062–1075. 10.1111/jcmm.12761 26769343 PMC4882989

[B62] Zheng B. Zhou Y. Zhang H. Yang G. Hong Z. Han D. (2017). Dl-3-n-butylphthalide prevents the disruption of blood-spinal cord barrier via inhibiting endoplasmic reticulum stress following spinal cord injury. *Int J Biol Sci* 13(12), 1520–1531. 10.7150/ijbs.21107 29230100 PMC5723918

